# On the Peculiar Effects Produced by Certain Odours

**Published:** 1810-09

**Authors:** R. Hall


### *On the peculiar Effects produced by certain Odours.*

By R. Hall, M. D.

VARIOUS cases of idiosyncracy, or peculiarity of temperament with respect to particular effluvia or odours, are recorded not only in the writings of physicians, but also in other publications. The effect produced by the odour of ipecacuanha on the lady of the late Dr. Buckham of Wooler, mentioned by Dr. Trotter in the last Number of the Medical and Physical Journal, I have myself oftener than once witnessed, while residing with that gentleman. So exquisitely sensible, indeed, was she to the effluvia of this substance, that I have known her subjected to dyspnœa, merely in consequence of pulvis ipecacuanha having been incautiously handled, though she was at that time in an adjoining apartment. It may not be here improper to remark, that though this lady was often unavoidably exposed to various medicinal effluvia, not only belonging, like ipecacuanha, to the class of odours termed *nauseosi*, but also to those denominated *tetri*, *ambrosiaci*, &c. yet from none of these did she ever, so far as I know, experience the slightest inconvenience. I have likewise heard of other females being affected in a similar manner by ipecacuanha; hence, I was led to consider this peculiarity to be so well known, that, like Dr. Trotter, I was surprised at Mr. Royston's conceiving he had been the first to remark it.

Many individuals, it is also well known, experience a sense of suffocation when a candle is imperfectly extinguished, particularly at night, after retiring to rest; while, on others, it produces no such effect. Nor are the respiratory the only organs which, in certain habits, are affected by particular effluvia. Thus the exhalations of camphor, though far from ungrateful to many, not unfrequently induce head-achs, particularly in females endowed with great nervous sensibility. The stomach is frequently known likewise to suffer, in some individuals, from the impression of particular effluvia on the olfactory nerves. A relative of my own was so sensibly affected by the odour of old ewe cheese, as to be seized with anorexia, nausea, and even sometimes vomiting, whenever he came within the sphere of its exhalations. An acquaintance, supposing that this might proceed rather from the sight than the smell of the substance in question, determined to resort resort to what he considered an experimentum crucis, in order to satisfy his doubts on the subject. With this view, he slipped into his pocket a piece of old cheese; but tho' the subject of his experiment was wholly unconscious of the action, the usual effects were in a short time produced by it.

While these, and many other similar facts, which are well known to physicians, serve to demonstrate the sympathetic connection that subsists between the lungs, the stomach, the brain, and the olfactory organ, they also shew us, that some odours or effluvia, though wholly innocuous to the generality of mankind, nevertheless prove highly injurious to others, in consequence of a peculiarity of habit, either innate or acquired.

*London, July* 26, 1810.

